# Index of Cough Symptoms

**Published:** 1882-05

**Authors:** 


### AN INDEX OF COUGH SYMPTOMS.

For some time past, Dr. G. H. Clark and the editor have been preparing an index of *all* symptoms of cough and expectoration which they could gather from reliable sources. The symptoms will be arranged alphabetically under the anatomical part in which they occur. The editors desire to make this index thoroughly reliable, and as complete as possible, they therefore solicit contributions from any physician who has reliable characteristic symptoms, hitherto unpublished. The index will be published in THE HOMŒOPATHIC PHYSICIAN—about eight pages per month, until completed—commencing in June or July. Each symptom is credited to the authority from whom it was obtained.

